# Dysregulated lncRNA-miRNA-mRNA Network Reveals Patient Survival-Associated Modules and RNA Binding Proteins in Invasive Breast Carcinoma

**DOI:** 10.3389/fgene.2019.01284

**Published:** 2020-01-15

**Authors:** Yu Dong, Yang Xiao, Qihui Shi, Chunjie Jiang

**Affiliations:** ^1^ Key Laboratory of Systems Biomedicine (Ministry of Education), Shanghai Center for Systems Biomedicine, Shanghai Jiao Tong University, Shanghai, China; ^2^ Institute for Diabetes, Obesity, and Metabolism, Perelman School of Medicine at the University of Pennsylvania, Philadelphia, PA, United States; ^3^ Division of Endocrinology, Diabetes, and Metabolism, Department of Medicine, Perelman School of Medicine at the University of Pennsylvania, Philadelphia, PA, United States

**Keywords:** lncRNAs, RNA binding protein, miRNAs, integrative analysis, invasive breast carcinoma, biomarker

## Abstract

Breast cancer is the most common cancer in women, but few biomarkers are effective in clinic. Previous studies have shown the important roles of non-coding RNAs in diagnosis, prognosis, and therapy selection for breast cancer and have suggested the significance of integrating molecules at different levels to interpret the mechanism of breast cancer. Here, we collected transcriptome data including long non-coding RNA (lncRNA), microRNA (miRNA), and mRNA for ~1,200 samples, including 1079 invasive breast carcinoma samples and 104 normal samples, from The Cancer Genome Atlas (TCGA) project. We identified differentially expressed lncRNAs, miRNAs, and mRNAs that distinguished invasive carcinoma samples from normal samples. We further constructed an integrated dysregulated network consisting of differentially expressed lncRNAs, miRNAs, and mRNAs and found housekeeping and cancer-related functions. Moreover, 58 RNA binding proteins (RBPs) involved in biological processes that are essential to maintain cell survival were found in the dysregulated network, and 10 were correlated with overall survival. In addition, we identified two modules that stratify patients into high- and low-risk subgroups. The expression patterns of these two modules were significantly different in invasive carcinoma versus normal samples, and some molecules were high-confidence biomarkers of breast cancer. Together, these data demonstrated an important clinical application for improving outcome prediction for invasive breast cancers.

## Introduction

In women, breast cancer is the most commonly diagnosed cancer and accounts for ~30% of new cancer diagnoses (Siegel et al., 2017). Great improvements have been achieved in diagnosis, surgery, and medical treatment for breast cancer in the past decades. From 1989 to 2016, the death rate for breast cancer dropped by 40% for female breast cancers in the United States. However, it has still been the second leading cause of cancer death in women in the last ten years (Siegel et al., 2017). Invasive breast carcinoma accounts for about 80% of breast cancer (Weigelt et al., 2008) and exhibits high heterogeneity in terms of morphology, clinical features, and prognosis (Milanovic et al., 2013), and the regulatory mechanisms at the genomic level still thus need to be unearthed.

Many studies have investigated the pathogenesis underlying breast cancer and have discovered diagnostic and prognostic markers. In 2006, a study reported altered expression patterns of microRNAs (miRNAs) during initiation and progression and their relationship with cancer diagnosis, staging, and prognosis (Calin and Croce, 2006). Another study investigated the expression of deregulated miRNAs in breast cancer and found correlations of altered miRNA expression with estrogen receptor expression, vascular invasion, and other clinicopathological characteristics (Iorio et al., 2005). Long non-coding RNAs (lncRNAs) represent a new class of non-coding RNAs that are at least 200 nucleotides in length and do not possess a clearly defined open reading frame (Ponting et al., 2009). lncRNAs are critical regulatory factors in cancer initiation and progression (Li and Chen, 2013; Yang et al., 2014). The lncRNA DSCAM-AS1 holds a central position in estrogen receptor (ER)-regulated breast cancer and modulates tamoxifen resistance and tumor progression (Niknafs et al., 2016). Another lncRNA, MAGI2-AS3, can target the Fas/FasL signaling pathway to suppress cell growth in breast cancer (Yang et al., 2018b). Furthermore, a 12-lncRNA signature has been proposed that can be used to identify breast cancer patients at high risk of tumor recurrence, which could be utilized in clinic (Zhou et al., 2016b). Recently, some studies have shown that post-transcriptional regulatory networks can be regulated by molecules at multiple levels (Wei et al., 2017; Liu et al., 2019). By constructing a ceRNA network, a 10-lncRNA signature has been proposed that classified patients into high- and low-risk subgroups with significantly different survival outcomes, highlighting the value of integrating data sets from multiple levels (Zhou et al., 2016a). Mir-21 and lncRNA AWPPH regulate cancer cell chemosensitivity and proliferation in triple-negative breast cancer (Liu et al., 2019). Mir-223 promotes breast cancer cell proliferation by targeting FOXO1 and provides a new potential tumor marker (Wei et al., 2017). The above results imply the significance of integrating molecules at different regulatory levels for interpreting the mechanism of breast cancer, especially in invasive breast carcinoma.

RNA-binding proteins (RBPs) are a type of proteins that bind RNA through its globular RNA-binding domains (RBDs) (Hentze et al., 2018). RBPs can bind mRNA, pre-rRNA, tRNA, small nuclear RNA (snRNA), small nucleolar RNA (snoRNA) and residual ncRNA (Gerstberger et al., 2014) and can alter the fate or function of the bound RNAs during post-transcriptional gene regulation (PTGR), which correlates with the stability, transport, localization, and degradation of different RNAs. They act as important participants in gene regulation (Nishida et al., 2017) and play an important role in maintaining genome integrity (Gerstberger et al., 2014). RBPs have been found to be closely related to many human diseases and to be involved in a wide range of biological processes, such as tumorigenesis, proliferation, development, and apoptosis, by interacting with mRNA (Frisone et al., 2015; Grammatikakis et al., 2017), microRNA (Ciafre and Galardi, 2013), and lncRNA (Luo et al., 2013; Schmitt and Chang, 2016). There are ~20,000 protein-coding genes in humans, and 7.5% of genes are involved in RNA metabolism by binding to RNA (Hentze et al., 2018). But only a few RBPs have received intensive study.

The Cancer Genome Atlas (TCGA) project was launched in 2005 and has accelerated the comprehensive understanding of cancer genomic profiles, thus improving diagnostic methods, therapy standards, and preventive strategies. TCGA has released thousands of high-throughput molecular profiles at different levels, which help researchers better understand cancer pathogenesis, diagnosis, and prognosis. In this study, we integrated the expression profiles of breast cancer at multiple levels (lncRNA, miRNA, and mRNA) across ~1,200 samples, including 1079 invasive breast carcinoma samples as well as 104 normal samples. We identified differentially expressed lncRNAs, miRNAs, and mRNAs and then constructing a lncRNA-miRNA-mRNA dysregulated network, which is a power-law, small-world network. RBPs were found in the dysregulated network, and some of them are related to overall survival time. In addition, two modules were identified and exhibited a correlation with the overall survival time. Further analysis showed that these modules have significantly different expression patterns in cancer versus normal samples. To better understand these two modules, we mined the literature for the molecules in each module and found that some molecules play important roles in breast cancer biology.

## Materials and Methods

### RNA-Seq Expression Data Sets and Pre-Processing

RNA-seq expression data sets of ~1200 patient samples were downloaded from TCGA (https://portal.gdc.cancer.gov/), comprising 1079 invasive breast carcinoma samples and 104 normal samples (**Table S1**). MRNA, lncRNA, and miRNA were included in each sample. Using Perl scripts, we combined ~1200 files into a single profile. The lncRNA expression profile was extracted from the profile based on the latest annotation from the Ensembl database. The biotypes of known lncRNAs are 3prime_overlapping_ncrna, ambiguous_orf, antisense, antisense_RNA, lincRNA, ncrna_host, non_coding, non_stop_decay, processed_transcript, retained_intron, sense_intronic, and sense_overlapping. The biotype of protein-coding genes is protein_coding. In total, 19951 mRNA, 15949 lncRNA, and 1881 miRNA were obtained from TCGA. Based on previously published papers (Yan et al., 2015; Li et al., 2018b; Pan et al., 2018), RNAs with expression 0 in more than 10% of normal samples were eliminated.

### Analysis of Differential Expression Between Breast Carcinoma and Normal Samples

Differentially expressed molecules were identified through the use of previously reported methods (Li et al., 2015; Li et al., 2018b). Firstly, RNAs were divided into two groups. RNAs with an expression level equal to 0 in <30% tumor samples were subjected to a t-test, and RNAs with an expression level equal to 0 in >30% tumor samples were subjected to a Fisher’s exact test. For the RNAs in the first group, RNAs with a fold change larger than 2 (or smaller than 0.5) and an adjusted p-value smaller than 0.01 were identified as differentially expressed. For RNAs in the second group, we determined their expression in binary fashion: ON (expressed, expression value larger than 0) and OFF (not detected, expression value equal 0). Firstly, the frequencies of ON and OFF in breast carcinoma and normal samples were calculated, respectively. RNAs expressed twice more frequently in cancer than in normal samples were marked as ‘ON in cancer’; otherwise, RNAs were marked as ‘OFF in Cancer.’ Then, for each RNA, the significance of the contingency between ON/OFF and cancer/normal status was calculated by Fisher’s exact test with adjustment for multiple testing *via* the Benjamini-Hochberg method. RNAs with FDR smaller than 0.01 was used. In total, 4269 differentially expressed protein-coding genes were identified, as well as 3057 differentially expressed lncRNAs and 367 differentially expressed miRNAs (**Tables S2**–**S5**). Validation of the differentially expressed RNAs was performed by extracting the expression values and normalized them based on Z-score. After that, based on the R package ‘pheatmap,’ samples were clustered using differentially expressed lncRNAs, miRNAs, and mRNAs, respectively. PCA was also used to cluster the samples.

### Ago CLIP-Seq-Supported miRNA Target Sites

miRNA target sites were predicted using a target prediction algorithm from miRanda (Betel et al., 2010) with the default parameters. 3’UTR was used to predict target sites for mRNA, while for lncRNA, the full length of the lncRNA transcript was used. It has been reported that miRNAs function in the form of ribonucleoprotein complexes, RISCs (RNA-induced silencing complexes) (Fabian et al., 2010), and Argonaute (AGO)-family proteins represent the best-characterized protein components and are central to RISC function (Eulalio et al., 2008; Chekulaeva and Filipowicz, 2009). Ultraviolet (UV) crosslinking and immunoprecipitation (CLIP) was used to identify specific protein-RNA interactions (Konig et al., 2012). Hence the function of the Argonate-RNA-miRNA complex can be verified through CLIP technology (Chou et al., 2013). Here we downloaded AGO 1/2 CLIP-Seq datasets from starBase v2.0 (Li et al., 2014a) and identified AGO binding sites to filter candidate miRNA target sites. A target was reserved only if it overlapped with at least one AGO binding site. In total, 41632 miRNA-lncRNA regulatory relationships were predicted, including 1176 lncRNAs and 2509 miRNAs, and 1247237 miRNA-mRNA regulatory relationships were predicted, including 18252 protein-coding genes and 2511 miRNAs (**Tables S6** and **S7**).

### Constructing the Dysregulated lncRNA-miRNA-mRNA Network

Based on the interactions of miRNA-lncRNA and miRNA-mRNA, we constructed an initial lncRNA-miRNA-mRNA network. A three-step filtering process was then performed: 1) RNAs that were not differentially expressed were filtered; 2) the expression of each RNA pair (miRNA-lncRNA or miRNA-mRNA) should be significantly correlated (p-value < 0.01 and |correlation coefficient| > 0.4) across samples based on Pearson correlation; 3) only miRNAs that were shared by both lncRNA and mRNA were considered. The dysregulated network was constructed based on 876 interactions and 539 differentially expressed molecules, including 75 miRNAs, 63 lncRNAs, and 401 protein-coding genes (**Table S8**). The network was visualized using Cytoscape (Shannon et al., 2003). CytoCluster (Li et al., 2017), a Cytoscape plugin for cluster analysis and visualization of biological networks, was used to identify modules, employing the graphically based IPC-MCE algorithm and adopting the default parameter values (0.6 as the Threshold).

### Survival Analysis

The clinical data of all of the breast cancer patients were downloaded from TCGA. Perl scripts were used to extract the information regarding days to last follow up and vital status (alive or dead) for each invasive breast carcinoma patient. For each module, the average value in each sample was used. Modules that relate to the overall survival were identified by clustering the samples into two classes based on K-means Clustering. An R package, ‘survival’ was then used to 1) construct a surv object using the function ‘Surv’ based on the status and time, 2) create fitted survival curves with the Kaplan-Meier algorithm, using the function ‘survfit’ based on the surv object and class label, and 3) test for a difference between the two survival curves using a log-rank test. P-value < 0.05 was set as the cutoff. All reported p-values were two-sided.

### Functional Enrichment Analysis

In order to investigate functional roles, GO and KEGG analyses were performed based on protein-coding genes in the network using the Database for Annotation, Visualization, and Integrated Discovery (DAVID, version 6.8) (Huang da et al., 2009; Huang et al., 2009). Cancer hallmarks related GO terms were identified by two previous studies (Subramanian et al., 2005; Plaisier et al., 2012). Additionally, PANTHER (Mi and Thomas, 2009) (https://reactome.org/) and REACTOME (Croft et al., 2011) (http://pantherdb.org/) pathway analysis were performed. To further investigate the functional roles, GAD, a database of genetic association data from complex diseases and disorders, was also used by DAVID (Huang da et al., 2009; Huang et al., 2009).

### Cancer Genes

Two cancer gene lists were used to further validate the roles in cancer. The first one was compiled by by Mertins et al. (2016), who collected 415 oncogenes and tumor suppressors from UniProt (https://www.uniprot.org/) and published papers. Another list of 524 genes that had been implicated in malignant transformation according to a catalog of somatic mutations in cancer (COSMIC, http://cancer.sanger.ac.uk/cancergenome/projects/census) was collected by Uhlen et al. (Uhlen et al., 2015). In total, 724 potentially cancer-related genes were used (**Table S9**).

## Results

### Differentially Expressed RNAs Distinguish Invasive Breast Carcinoma From Normal Tissues

We acquired the expression profiles of mRNA, lncRNA, and miRNA from TCGA, which contains 1183 samples, comprising 1079 invasive breast carcinoma samples and 104 normal samples (**Table S1**). Differentially expressed molecules were identified using the method detailed in Li et al. (2018b). RNAs with an expression level equal to 0 in <30% tumor samples were subjected to t-test, and RNAs with an expression level equal to 0 in >30% tumor samples were subjected to Fisher’s exact test (see Methods). In total, 4269 protein-coding genes that were differentially expressed between invasive breast carcinoma and normal samples were identified, including 2349 up-regulated and 1920 down-regulated genes (**Tables S2** and **S3**). For lncRNAs, 3057 differentially expressed molecules were identified, of which 2033 were up-regulated and 1024 were down-regulated (**Tables S2** and **S4**). Additionally, 367 differentially expressed miRNAs were identified. 152 miRNAs were up-regulated, and 215 were down-regulated (**Tables S2** and **S5**).

**Figure 1:**
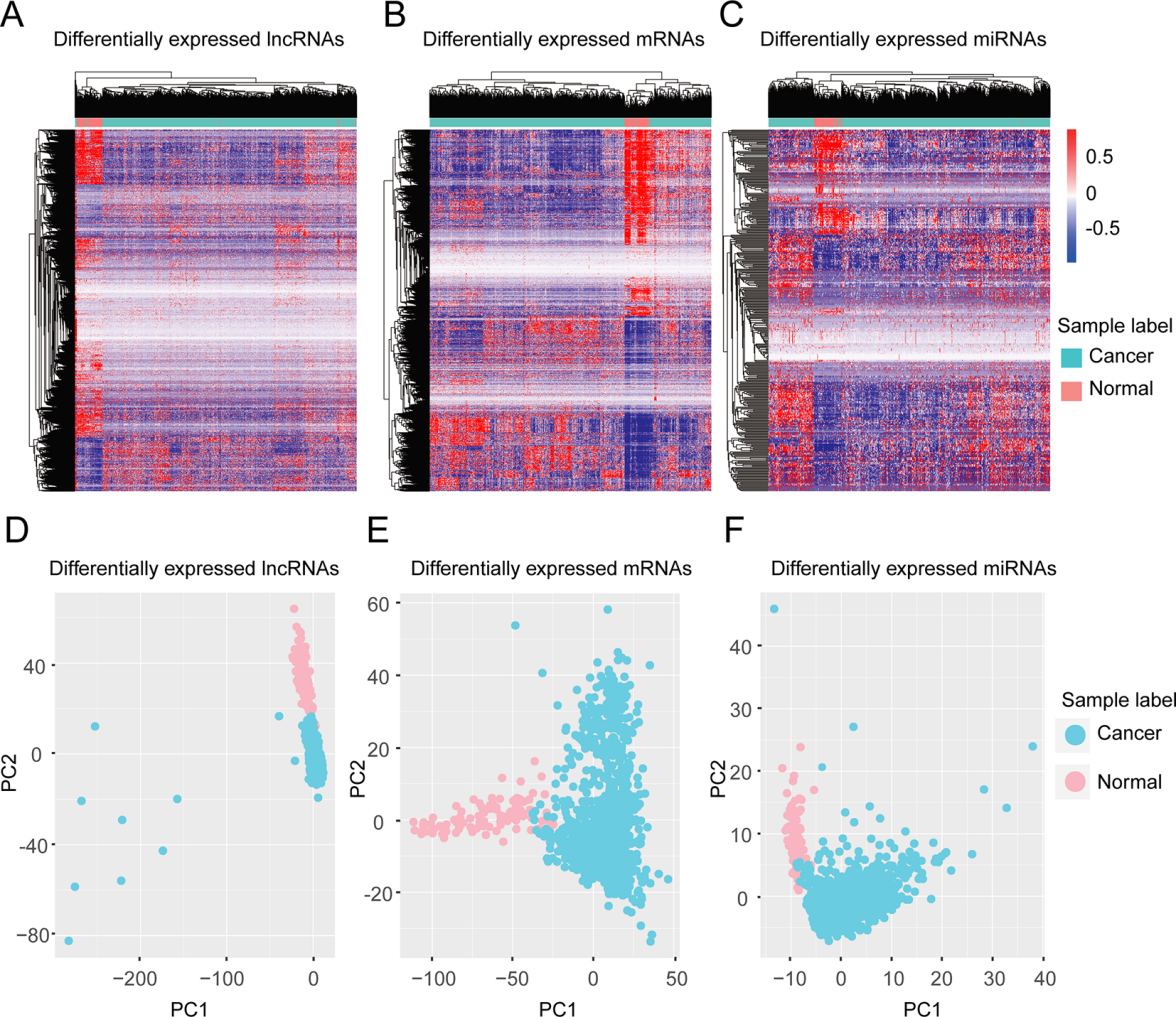
Clustering based on differentially expressed molecules. Unsupervised hierarchical clustering of all samples based on differentially expressed lncRNAs **(A)** protein-coding genes **(B)** and miRNAs **(C)**. The unsupervised hierarchical clustering was performed using an R package, ‘pheatmap’ with the default distance setting, Euclidean distance. **(D–F)** PCA analysis based on differentially expressed lncRNAs **(D)**, protein-coding genes **(E)**, and miRNAs **(F)**. PCA analysis was performed by the R function ‘prcomp’.

We validated our differentially expressed molecules by performing unsupervised hierarchical cluster analyses for the 1179 invasive breast carcinoma samples and 104 normal samples using the R package ‘pheatmap’ with the default distance. The invasive breast carcinoma samples were clearly distinguished from normal samples in terms of differentially expressed lncRNAs, protein-coding genes, and miRNAs, respectively (**Figures 1A**–**C**). To further check these differentially expressed molecules, principle component analysis (PCA) analyses were performed using the R function ‘prcomp.’ Consistent with the unsupervised hierarchical clustering, the first two principal components could distinguish the tumor samples from normal samples (**Figures 1D**–**F**).

### The Dysregulated Network Is a Biological Network Performing Housekeeping and Cancer-Related Functions

**Figure 2:**
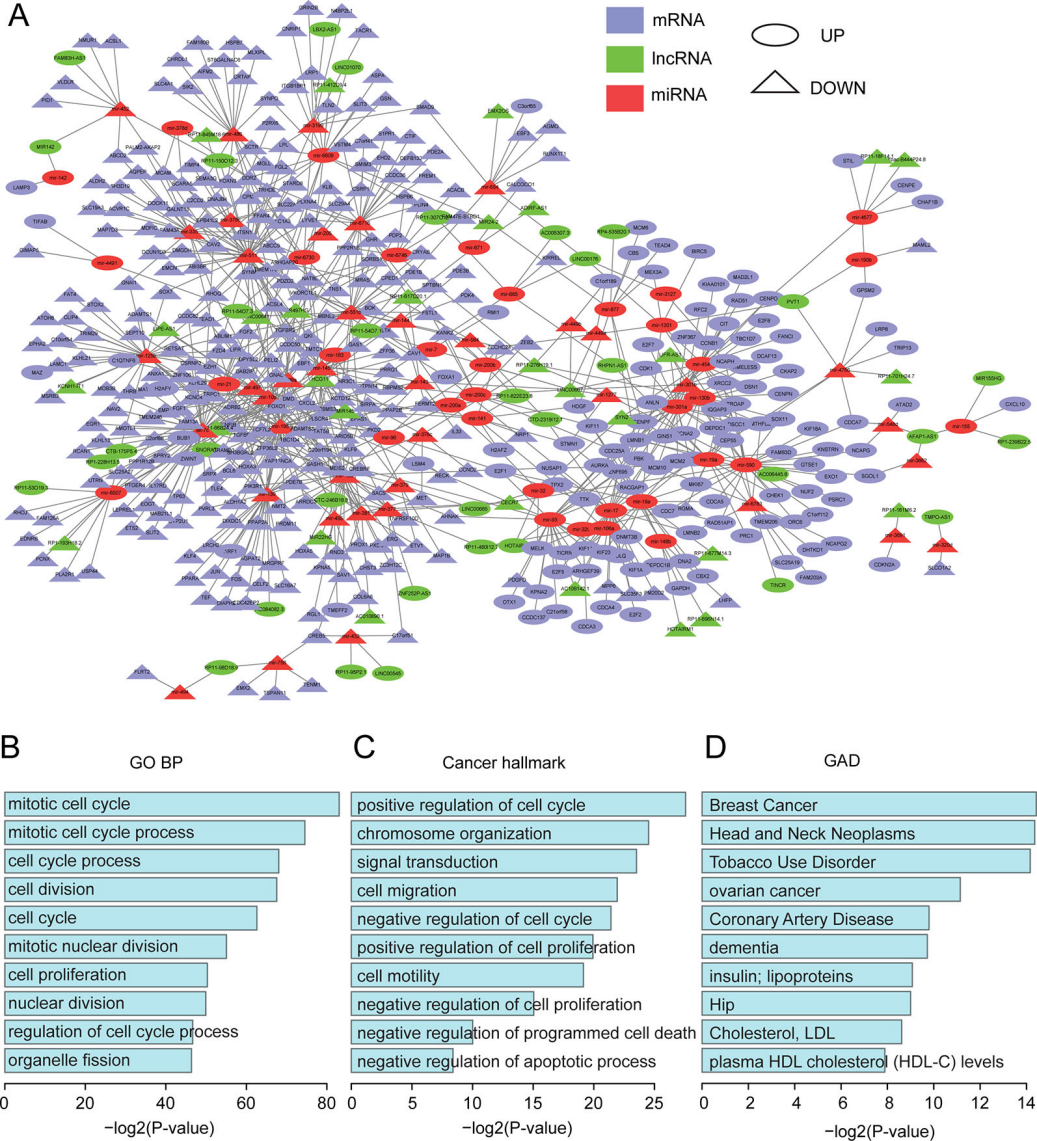
Functional analysis for the dysregulated network. **(A)** The dysregulated lncRNA-miRNA-mRNA network. The network was visualized using Cytoscape. **(B)** The top 10 enriched GO terms. **(C)** The top 10 enriched cancer hallmark-related GO terms. **(D)** The top 10 enriched GAD terms.

All of the differentially expressed molecules mentioned above were used to construct the dysregulated network. We predicted miRNA target sites for all protein-coding genes and lncRNAs based on the algorithm from miRanda (Betel et al., 2010), using the default parameters (see Methods). It has been reported that miRNA functions in the form of ribonucleoprotein complexes, RISCs (RNA-induced silencing complexes) (Fabian et al., 2010), and Argonaute (AGO)-family proteins represent the best-characterized protein components and are central to RISC function (Eulalio et al., 2008; Chekulaeva and Filipowicz, 2009). Ultraviolet (UV) crosslinking and immunoprecipitation (CLIP) was used to identify specific protein-RNA interactions (Konig et al., 2012). Hence, the function of the Argonate-RNA-miRNA complex can be verified through CLIP technology. Candidate miRNA and target site pairs were filtered by the AGO 1/2 CLIP-seq data from starBase (Li et al., 2014a) (see *Materials and Methods*). A total of 41632 interactions, including 2509 miRNAs and 1176 lncRNA targets, were predicted as well as 1247237 interactions between 2577 miRNAs and 18252 protein-coding genes (**Tables S6** and **S7**). Next, based on the three-step filtering process (see *Materials and Methods*), a dysregulated network was constructed from 876 interactions and 539 differentially expressed molecules, including 75 miRNAs, 63 lncRNAs, and 401 protein-coding genes (**Figure 2A**, **Table S8**).

It has been shown that many biological networks are small-world networks (Latora and Marchiori, 2001; Wagner and Fell, 2001), which have also been reported to be scale-free networks (Amaral et al., 2000). We tested whether our dysregulated network is a scale-free network by analyzing the degree distribution, which is one of the most important characteristics of a scale-free network and is defined as the number of edges incident to a node. As shown in **Figure S1A**, more than 88% of nodes had less than five edges, whereas only 6% of nodes had more than 10 edges. It fitted a power-law distribution with *R* ^2^ = 0.86 and correlation = 0.99, suggesting that our network is a scale-free network (Barabasi, 2009). In addition, most of the shortest paths were between 4 and 6 (**Figure S1B**), which is consistent with the property of a small-world network. Closeness is a measure of how close an individual is to other individuals in a network (Borgatti, 1995; Costenbader and Valente, 2003). The more central a node is, the closer it is to all other nodes. As shown in **Figure S1C**, the majority of nodes were highly central. Together, these data revealed that our dysregulated network is a scale-free and small-world network, indicating that our network is a canonical biological network.

The functions of the dysregulated network were investigated by using the protein-coding genes in this network to perform functional enrichment analysis (see Methods). All top-ten enriched gene ontology (GO) terms were related to cell cycle, mitotic nuclear division, and nuclear division (**Figure 2B**). These were all housekeeping functions for maintaining cell survival. We further acquired all of the housekeeping genes identified by Jiang et al. (2018) and found that 84 (21%) protein-coding genes in our dysregulated network were housekeeping genes (**Table S10**). In addition, based on a previous study (Salem et al., 2016), we obtained a list of GO terms related to hallmarks of cancer and found that these terms were also enriched in our network (**Figure 2C**). For example, signal transduction (GO:0007165) and positive regulation of cell proliferation (GO:0008284) are Self Sufficiency in Growth Signals, while negative regulation of cell proliferation (GO:0008285) and negative regulation of cell cycle (GO:0045786) are Insensitivity to Antigrowth Signals. Taken together, our dysregulated network demonstrated important and functional roles.

Moreover, we performed pathway enrichment analyses using three different pathway databases, the Kyoto Encyclopedia of Genes and Genomes (KEGG), PANTHER (Mi and Thomas, 2009), and REACTOME (Croft et al., 2011). For the KEGG pathway, housekeeping and cancer-related functions were again enriched (**Figure S2A**). The housekeeping functions were cell cycle and axon guidance, and the cancer-related functions were pathways in cancer, Melanoma, Colorectal cancer and Prostate cancer (**Figure S2A**). For the PANTHER and REACTOME pathway databases, most of the top terms were housekeeping functions (**Figures S2B**, **C**).

To further validate their important roles in cancer, we obtained 415 oncogenes and tumor suppressors from Mertins et al. (2016) and 524 genes that have been implicated in malignant transformation from Uhlen et al. (2015). In total, 724 cancer genes were used (**Table S9**). 656 of them were expressed in our dataset, and 31 were in our network (**Figure S2D**). Based on a hypergeometric test, the p-value was 7.46E-05, which suggested that our dysregulated network was significantly enriched in cancer-related genes. We further performed functional enrichment analysis using DAVID (Huang da et al., 2009; Huang et al., 2009) based on the Genetic Association Database (GAD), which is a database of genetic association data from complex diseases and disorders. Surprisingly, breast cancer was the most enriched term (**Figure 2D**), which corroborated the important roles of our dysregulated network in cancer biology.

### RBPs in Our Dysregulated Network

**Figure 3:**
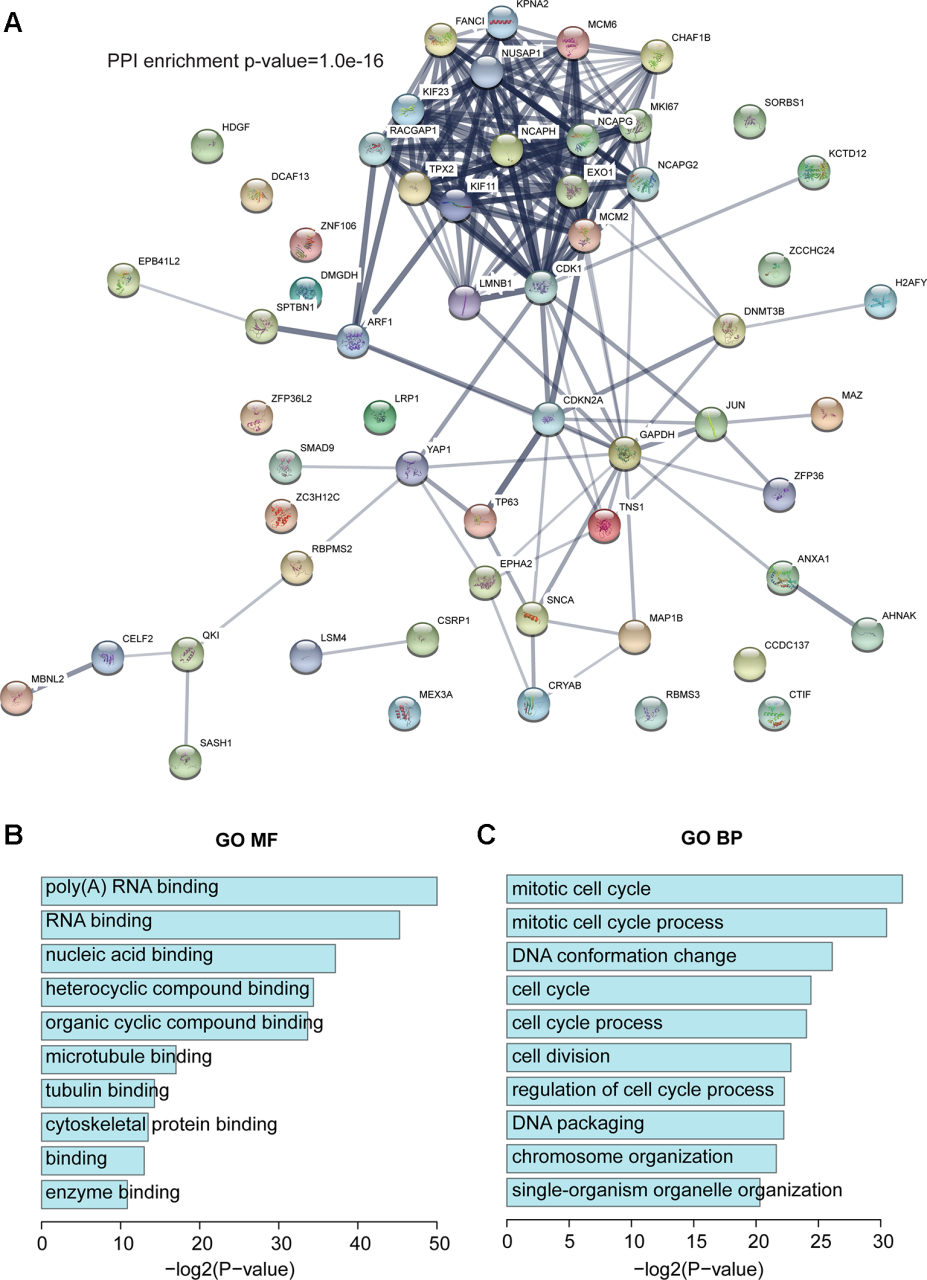
RBPs in the dysregulated network. **(A)** Protein–protein interaction (PPI) network of RBPs based on STRING. **(B)** The top 10 enriched GO MF terms. **(C)** The top 10 enriched GO BP terms. BP, biological process.

Next, we investigated the RBPs in our dysregulated network. Based on published papers (Cook et al., 2011; Gerstberger et al., 2014; Fredericks et al., 2015; Hentze et al., 2018), 58 RBPs were found in our dysregulated network, of which 28 were upregulated and 30 were downregulated (**Table S11**). To improve our understanding of the roles of RBP in invasive breast carcinoma, STRING (https://string-db.org/) was used to construct a protein–protein interaction (PPI) network (**Figure 3A**). Random networks of the same size were generated by STRING, which was used to assess whether the given network had more internal interactions than would be expected for a random set of the same size. A small PPI enrichment p-value indicates that the nodes are not random and that the observed number of edges is significant. Based on STRING, the PPI enrichment p-value was 1.0e-16, which means that these RBPs have more interactions than would occur in a random set. This enrichment indicated that these RBPs are at least partially biologically connected as a group. The GO analysis showed that all top 10 molecular function (MF) terms were binding-related functions and the top two were poly(A) RNA binding and RNA binding, which further confirms that they are RBPs (**Figure 3B**), and that these RBPs are involved biological processes that are essential to maintain cell survival like cell cycle, cell division, DNA packaging, and chromosome organization (**Figure 3C**). Moreover, GAD enrichment analysis was also performed, and it is worth noting that breast cancer was again the most enriched term (**Figure S3**).

To investigate whether these RBPs were associated with prognosis in invasive breast carcinoma patients, the overall survival for each RBP was calculated using the R package ‘survival’ (see Methods). Ten RBPs (CDKN2A, DCAF13, DNMT3B, EXO1, FANCI, KPNA2, RACGAP1, SORBS1, TP63, and ZNF106) were significantly associated with overall survival, including seven upregulated and three downregulated RBPs (**Figures S4** and **S5**). Notably, some were reported to play roles in breast cancer (see Discussion). Overexpression of DCAF13, DNMT3B, KPNA2, EXO1, FANCI, RACGAP1, and ZNF106 in invasive breast carcinoma patients showed poor survival, while overexpression of CDKN2A, SORBS1, and TP63 showed better survival (**Figures S4** and **S5**).

### Modules in the Dysregulated Network Relate to the Survival of Invasive Breast Carcinoma Patients

**Figure 4:**
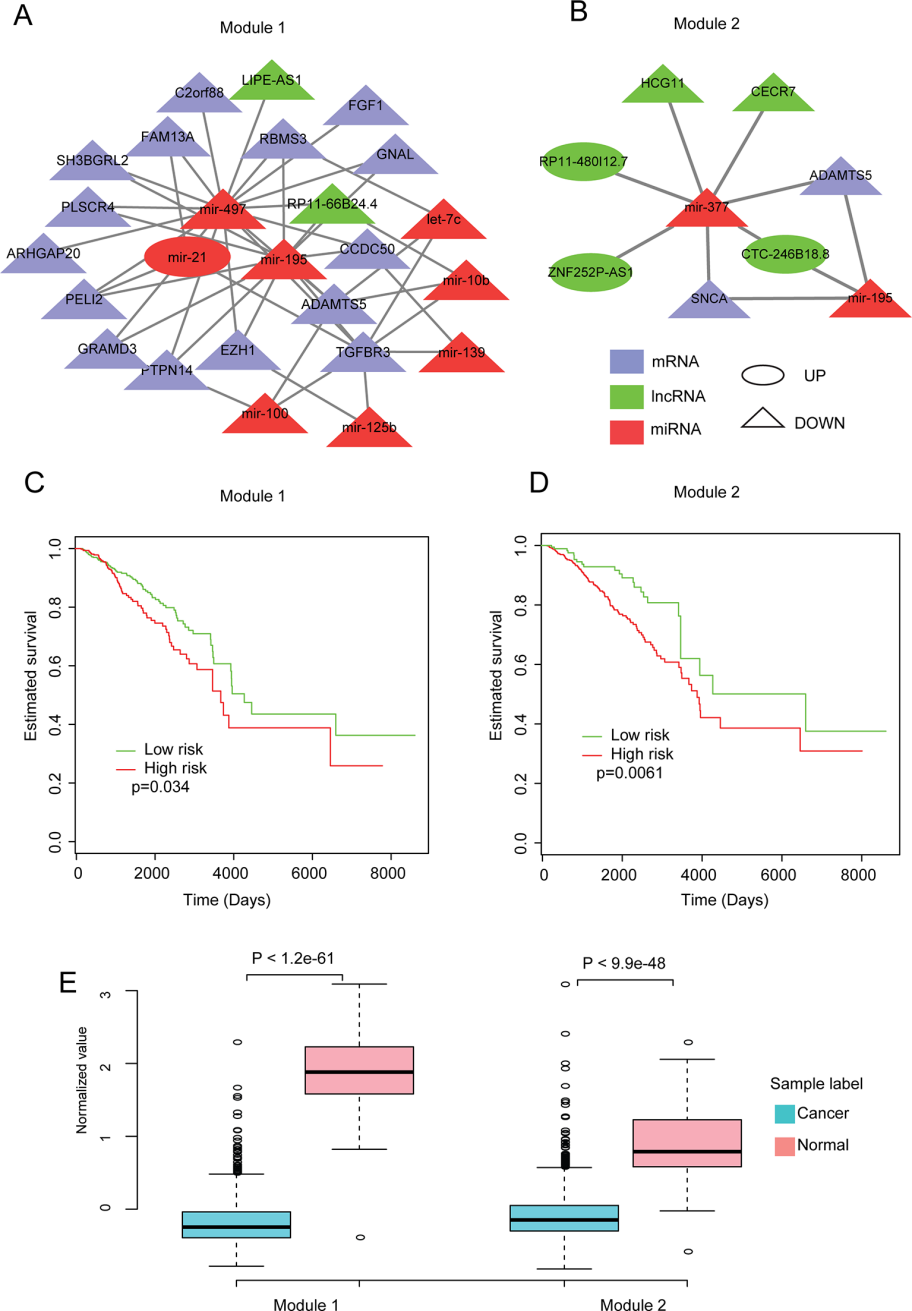
Analysis of modules identified from the dysregulated network. **(A**, **B)** The two modules identified from the dysregulated network using Cytoscape with default parameters. **(C**, **D)** Kaplan-Meier plot of survival for these two modules. **(E)** Expression patterns of the modules in normal and cancer samples. The average expression value of each molecule crossing all normal/cancer samples was used.

To further investigate the roles of our dysregulated network, CytoCluster (Li et al., 2017), a Cytoscape plugin for cluster analysis and visualization of biological networks, was used to identify modules (see Methods). Subsequently, to explore the relationship between the modules and the prognosis of patients with invasive breast carcinoma, the overall survival for each module in invasive breast carcinoma patients was investigated (see Methods). We found that two modules were significantly (p < 0.05) correlated with overall survival (**Figures 4A–D**). Moreover, their expression patterns in normal and invasive breast carcinoma samples were assessed. These two modules showed significant differences in expression patterns between normal and invasive breast carcinoma samples (**Figure 4E**). Both showed significantly lower expression in invasive breast carcinoma samples, indicating that lower expression of these modules contributes to the development of invasive breast carcinoma.

In addition, to further investigate the functions of these two modules in breast cancer, literature-mining was used for the molecules in each module. Module 1 had 25 nodes, including eight miRNAs, two lncRNAs, and 15 protein-coding genes. Twenty-two of the molecules, including all of the miRNAs, have been shown to play important roles in breast cancer. For example, mir-195 inhibits tumor growth and metastasis in breast cancer cells (Singh et al., 2015; Wang et al., 2016c). Mir-497 contributes to cell proliferation, migration, and invasion of estrogen receptor alpha-negative breast cancer by targeting estrogen-related receptor alpha (Han et al., 2016; Wu et al., 2016b). TGFBR3 inhibits breast cancer progression through TGF-beta signaling (Lee et al., 2010). In addition, other molecules in module 1 such as ADAMTS5 (Fontanil et al., 2017), ARHGAP20 (Asaduzzaman et al., 2017), C2orf88 (Lo et al., 2015), EZH1(Liu et al., 2012), FAM13A (Goto-Yamaguchi et al., 2018), FGF1 (Slattery et al., 2013), GNAL (Yi et al., 2009), GRAMD3 (Boiles et al., 2015), PELI2 (Zang et al., 2017), PLSCR4 (Sahay et al., 2015), PTPN14 (Belle et al., 2015), RBMS3 (Zhu et al., 2019a), SH3BGRL2 (Alexe et al., 2007; Wen et al., 2018), let-7c (Fu et al., 2017), mir-100 (Jiang et al., 2016b), mir-10b (Wang et al., 2016b), mir-125b (Wang et al., 2019a), mir-139 (Dai et al., 2017), and mir-21 (Yan et al., 2008; Yanwirasti and Arisanty, 2017; Zhu et al., 2019b) have been reported to play important roles in breast cancer. Module 2 had nine nodes, including two miRNAs, five lncRNAs, and two protein-coding genes. Five of these molecules have been shown to play important roles in breast cancer. For example, Wang et al., (2019b) reported that overexpression of miR-377 correlates with better prognosis in triple-negative breast cancer. ADAMTS-5 may alter the cellular microenvironment, affecting the balance between protumor and antitumor effects (Fontanil et al., 2017). SNCA is the hub gene and is involved in promoting tumor invasion in breast cancer (Serra-Musach et al., 2012; Dang et al., 2016). Besides, molecules in module 2 like the lncRNAs (HCG11) (Liu et al., 2016) and mir-195 (Singh et al., 2015; Wang et al., 2016c) have also been reported to play roles in breast cancer. We also performed key driver analysis (KDA) (Bin Zhang , 2013) to identify key drivers in our network, and all of the miRNAs from our two modules were identified as key drivers. All of these results imply the important roles and vital functions of these two modules in breast cancer biology.

## Discussion

Breast cancer is a leading type of cancer in women worldwide (Stewart and Wild, 2014). Many improvements have been made in diagnostic techniques, surgical skills, and medical treatments relating to breast cancer in the past decades. However, it still caused 522,000 deaths in 2012 (Stewart and Wild, 2014). It is imperative to improve the diagnosis and treatment of breast cancer further. Therefore, the identification of cancer-related molecules and the exact regulatory mechanism of breast cancer initiation and development are attracting increasing attention.

It has been reported that lncRNAs and miRNAs play important roles in breast cancer, as do protein-coding genes (Cizkova et al., 2013; Li et al., 2014b; Kim et al., 2015; Yang et al., 2018b). Here we integrated the expression data of lncRNA, miRNA, and protein-coding genes based on ~1200 invasive breast carcinoma and normal samples from TCGA. A total of 4269 differentially expressed protein-coding genes, 3057 differentially expressed lncRNAs, and 367 differentially expressed miRNAs were identified. Based on unsupervised hierarchical clustering and PCA, the samples from invasive breast cancer were distinguished from the normal samples. To construct a dysregulated network, we predicted miRNA targets using an algorithm from miRanda (Betel et al., 2010) with the default parameters. As mature miRNA is part of an active RNA-induced silencing complex (RISC) (Rana, 2007) and the Ago family is central to RISC function (Tang, 2005), AGO CLIP-Seq data were applied to achieve highly convincing miRNA targets. Based on the differentially expressed lncRNAs, miRNAs, and protein-coding genes, an initial dysregulated lncRNA-miRNA-mRNA network was built. After three-step filtering, the final network was constructed, consisting of 876 interactions and 539 differentially expressed molecules.

Next, we analyzed this network through different aspects—the distribution of degree, shortest path, and closeness centrality—which showed that the dysregulated network is a scale-free, small-world network and a meaningful biological network. To further understand the function of the dysregulated network, functional enrichment analysis was performed. The top-10 GO terms showed housekeeping functions in our network. Furthermore, terms related to cancer hallmarks were also found, based on a previous study (Salem et al., 2016). Enrichment analysis with three different pathway databases supported the housekeeping and cancer-related functions in our dysregulated network. Based on two previous studies, 716 potential cancer genes were obtained, and further analysis showed enrichment in these cancer-related genes. Furthermore, we found that breast cancer was the most enriched term based on GAD, suggesting the important role of our dysregulated network in cancer biology.

It was known that RBPs play a central role in the regulation of gene expression, and dysregulated expression of RBPs has been related to the development of cancers (Galante et al., 2009; Bebee et al., 2014; Wang et al., 2015; Correa et al., 2016). In the present study, we identified 58 RBPs in our dysregulated network, and these were confirmed by GO BP analysis. These RBPs are involved in biological processes that are essential to maintaining cell survival. Based on STRING, we found that these RBPs had more interactions among themselves than what would be expected, indicating that they are at least partially biologically connected. Interestingly, GAD enrichment analysis again showed that breast cancer was the most enriched term. In addition, 10 RBPs were found to be associated with the overall survival of invasive breast carcinoma patients, which suggested that they might be associated with tumor progression, invasion, and aggressiveness. Indeed, some have been reported to play roles in breast cancer. TP63 is a sequence-specific DNA binding transcriptional activator or repressor (Zhou et al., 2016b). In breast cancer, high expression of TP63 coupled with STAT6 has been shown to be associated with longer metastasis-free survival, indicating that TP63 could be involved in inhibiting the migration of breast cancer cells (Papageorgis et al., 2015). By silencing TP63 expression, breast cancer cells acquired increasing resistance to cisplatin, suggesting its role in drug reaction (Mendoza-Rodriguez et al., 2019). SORBS1 is an adaptor protein, and its overexpression inhibits the invasive capacity of tumor cells in breast cancer patients. Silencing SORBS1 promoted EMT and weakened chemotherapy sensitivity (Song et al., 2017). DCAF13, located in chromosome 8q22.3, has been shown to be amplified in breast cancer. Overexpression of DCAF13 was associated with worse prognosis and might be involved in regulating cell cycle progression (Chin et al., 2007; Cao et al., 2017). By targeting DNMT3b, miR-221 became involved in tumorigenicity through regulating the stemness of breast cancer cells (Roscigno et al., 2016). Additionally, DNMT3B helped maintain the CAF function of promoting breast cancer malignance (Tang et al., 2019). RBPs are important in tumor development, and their role still needs to be explored more.

Using CytoCluster (Li et al., 2017), we identified modules that were significantly related to the overall survival time. These two modules had significantly different expression patterns in cancer and in normal samples. Moreover, the literature mining revealed that some molecules in each module play important roles in breast cancer. In module 1, there were 25 nodes, including eight miRNAs, two lncRNAs, and 15 mRNAs. It had been reported that the upregulation of mir-497 inhibited cell proliferation, migration, and invasion in breast cancer (Han et al., 2016; Wang et al., 2016a; Wu et al., 2016b) and that mir-195 inhibited tumor growth, invasion, and metastasis by targeting other RNAs in breast cancer (Singh et al., 2015; Wang et al., 2016c). Importantly, mir-497 and mir-195 were the hub nodes in this module, indicating their essential role in the module. However, these two miRNAs were down-regulated in breast cancer, which means that the inhibition was lost, contributing to the development of breast cancer. Consistent with the tumor-suppressive role of these two miRNAs, TGFBR3 was reported to suppress breast cancer progression through TGF-beta signaling (Lee et al., 2010), and RBMS3 and PTPN14 were also shown to play roles in inhibiting metastasis (Belle et al., 2015; Zhu et al., 2019a). These data imply that the function of module 1 may be to inhibit cancer progress and metastasis and that these functional miRNAs may affect breast cancer through TGFBR3, RBMS3, and PTPN14. Additionally, LIPE-AS1 (lncRNA), RP11-66B24.4 (lncRNA), and CCDC50 (mRNA) have not been reported in BRCA, but LIPE-AS1 interacted with miRNA-497 and slightly correlated with overall survival (p = 0.075) and both RP11-66B24.4 and CCDC50 are regulated by the two hub miRNAs, which suggested that they might act as main or auxiliary regulators in the progression and metastasis of BRCA. In module 2, mRNA ADAMTS5 was reported to play roles during migration and invasion in breast cancer (Fontanil et al., 2017). It also functions as a tumor suppressor by inhibiting migration, invasion, and angiogenesis in human gastric cancer (Huang et al., 2019). Besides, two other studies have shown that the upregulation of ADAMTS5 promotes progression in colorectal cancer and drives metastasis in colon and non-small cell lung cancer (Gu et al., 2016; Yu et al., 2016). Another mRNA, SNCA, was also reported to be involved in tumor development by inhibiting invasion and inducing apoptosis (Li et al., 2018c; Yan et al., 2018). Thus, the function of module 2 might relate to cancer progression and survival. Previous studies have shown that miRNAs may function as tumor suppressors or oncogenes in tumor development, invasion, and metastasis. In module 2, mir-377 is the hub node and may be the core molecule involved in breast cancer due to its interactions with other molecules. Moreover, mir-377 has been reported to inhibit proliferation and metastasis in gastric cancer and pancreatic cancer (Chang et al., 2016; Wang et al., 2017). mir-195 was also important in BRCA, as it was shown that mir-195 could inhibit the invasion and metastasis of breast cancer (Singh et al., 2015; Wang et al., 2016c). lncRNA CECR7 interacts with mir-377 and had been reported to play a role in hepatocellular carcinoma (Zhang et al., 2015). In addition to the molecules reported to play roles in breast cancer, some novel candidate biomarkers, which may also be important to breast cancer, were found, but more evidence is needed in future.

Many studies have performed integrative analyses of TCGA breast cancer data through networks. For example, Yin et al. (2016) focused on identifying miRNA-mRNA pairs and constructed a miRNA target network in invasive breast carcinoma. Li et al. (2018a) found that some of the correlations between microRNA and target genes declined in cancer compared to normal across multiple cancers. Wu et al. (2016a) found two kinds of lncRNA-mRNA co-expression patterns: 1) correlations between lncRNA-mRNA in cancer were reversed compared to normal; 2) correlations between lncRNA-mRNA in cancer were similar to normal. Xiao et al. (2018) compared the differential genes between ER+ and ER- and constructed a ceRNA network and found that some molecules correlated with prognosis. Yang et al. (2018a) compared the differentially expressed genes in Triple-Negative Breast Cancer and also constructed a ceRNA network. Some molecules correlated with prognosis were identified and validated by qRT-PCR. Sun et al. (2019) identified eight lncRNAs as the prognosis signature for breast cancer using a ceRNA and WGCNA network. Gao et al. (2019) built a ceRNA and found some prognosis-related molecules (four lncRNAs, two miRNAs, and two mRNAs). Most studies built a ceRNA network, which contains molecules that are not differentially expressed. However, the integrated dysregulated network in this study consists of differentially expressed lncRNAs, miRNAs, and mRNAs only, and we identified RBPs and modules that can stratify patients into high- and low-risk subgroups. Moreover, each module not only relates to prognosis but also contains RNAs that have been reported to play roles in breast cancer.

It is well known that the expression of non-coding RNAs is highly tissue- and cell-type specific, providing important clues about their specific functions in response to contextual demands (Mercer et al., 2008; Cabili et al., 2011; Jiang et al., 2016a). Here, we identified patient survival-associated modules including non-coding RNAs in invasive breast carcinoma, and this interpretation was supported in many ways. All molecules in the modules were differentially expressed in invasive breast carcinoma, indicating the potential roles of these molecules. The modules came from a scale-free biological network that performs functions that are related to housekeeping and are cancer hallmarks. More importantly, these two modules were significantly correlated with overall survival. Moreover, many papers have shown clues that molecules in our networks play roles in the progression of breast cancer, and KDA analysis also showed that the molecules in our networks are key drivers. Based on these strands of evidence, our results are credible. However, there are limitations to this study. Firstly, it is a network-based study. Secondly, our study is only based on bioinformatics analysis. Experiments are needed to support the identifications of functional roles.

## Conclusions

In summary, using a network-based strategy, we provided a framework integrating miRNAs, mRNAs, and lncRNAs that are differentially expressed in breast cancer to identify biomarkers. Although further validation is still needed to support the potential roles of the RBPs and two modules, many strands of evidence show the correlations between our two modules and breast cancer. Overall, our dysregulated network provides new insights into outcome prediction for invasive breast cancers.

## Data Availability Statement

Publicly available datasets were analyzed in this study. This data can be found here: https://portal.gdc.cancer.gov/.

## Author Contributions

CJ and YD were responsible for the statistical analysis, contributed to the acquisition of data, and were major contributors to writing the manuscript. QS and YX assisted with data analysis and revised the manuscript. All authors read and approved the final manuscript.

## Conflict of Interest

The authors declare that the research was conducted in the absence of any commercial or financial relationships that could be construed as a potential conflict of interest.
