# Supplementary material for: Dysregulated lncRNA-miRNA-mRNA Network Reveals Patient Survival-Associated Modules and RNA Binding Proteins in Invasive Breast Carcinoma
Source: Front Genet. 2020 Jan 15;10:1284. doi: 10.3389/fgene.2019.01284 (PMC6975227; doi:10.3389/fgene.2019.01284)
Supplement: Supplementary file 2 [file Image_1.pdf]

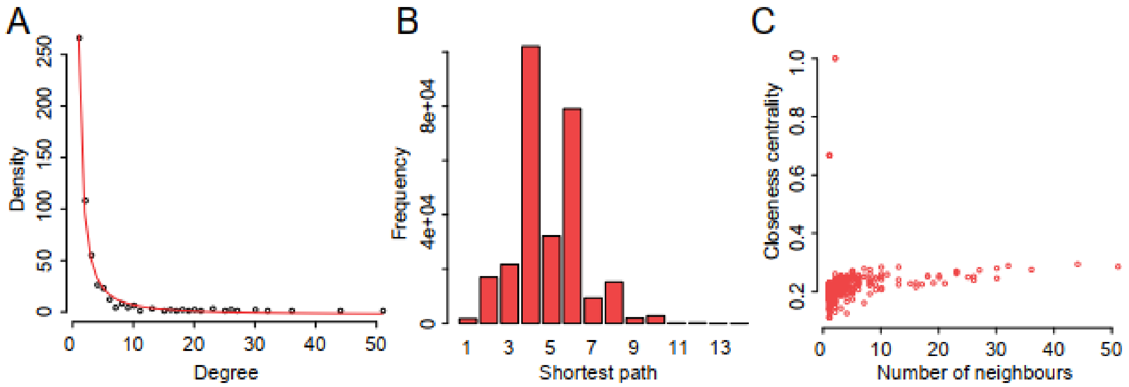

Figure S1. Network analysis. (A) The distribution of degree. The fitting line was obtained by R function 'nls. (B) The distribution of shortest path. (C) Closeness centrality of the dysregulated network.

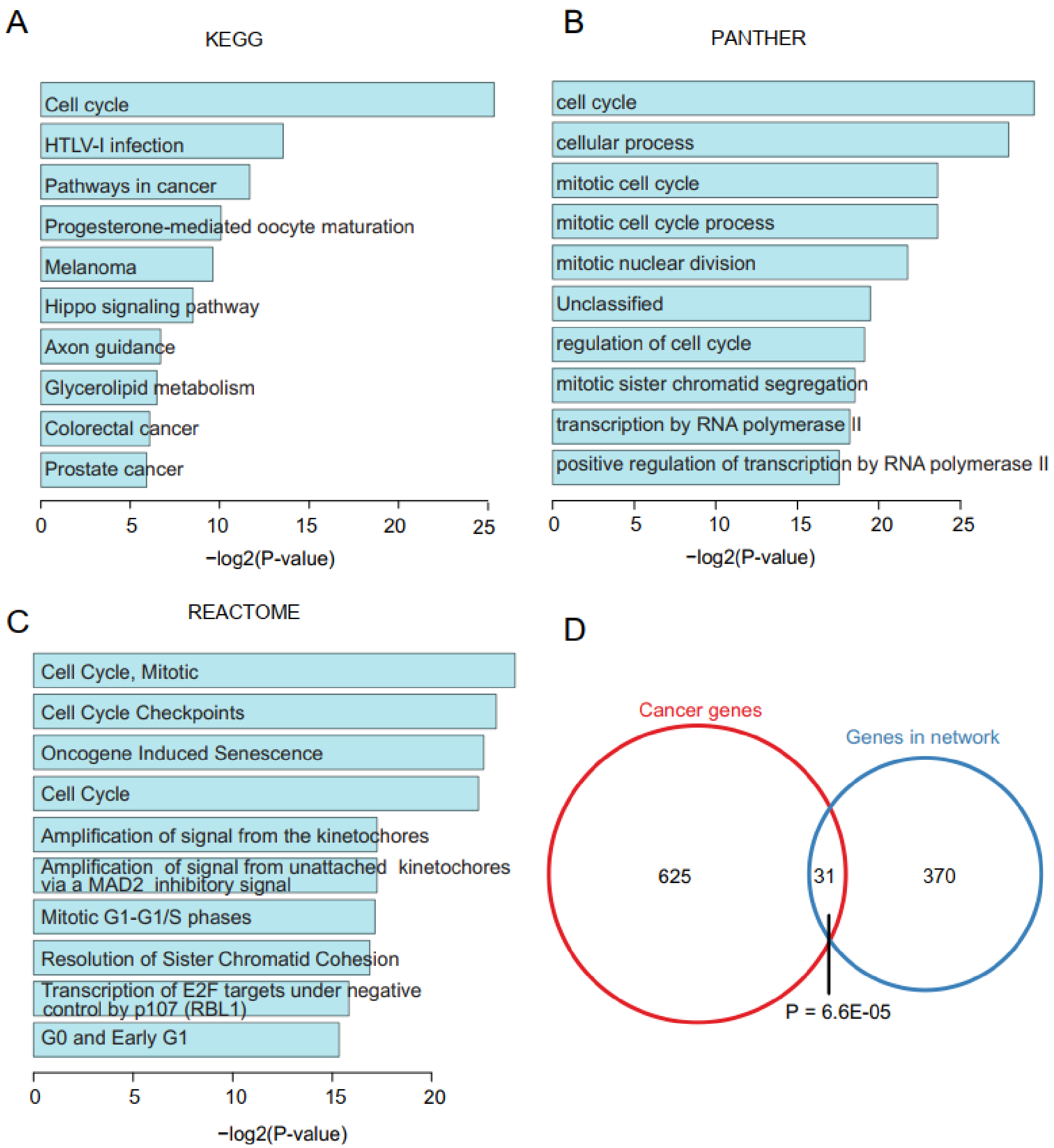

Figure S2. Functional analysis for the dysregulated network. (A) The top 10 enriched KEGG pathways (B) The top 10 enriched PANTHER pathways. (C) The top 10 enriched REACTOME pathways. (D) The overlap between the protein-coding genes in the dysregulated network and cancer genes. All expressed protein-coding genes were used as the background.

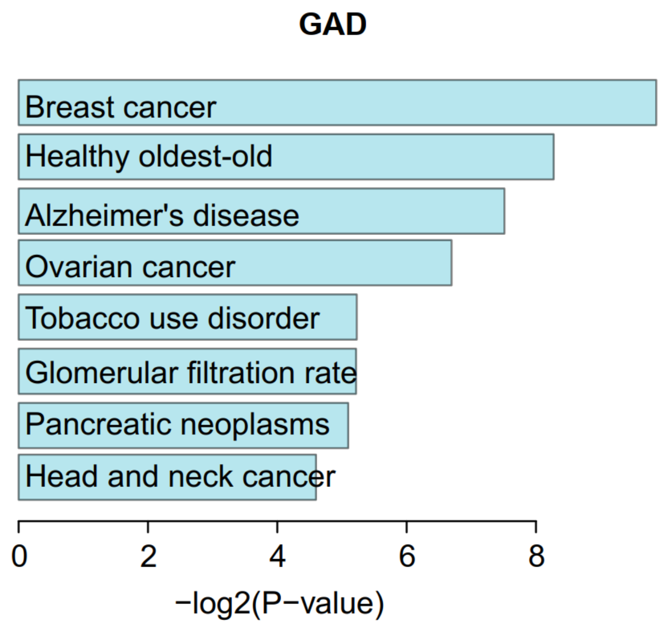

Figure S3. GAD enrichment for RBPs in the dysregulated network.

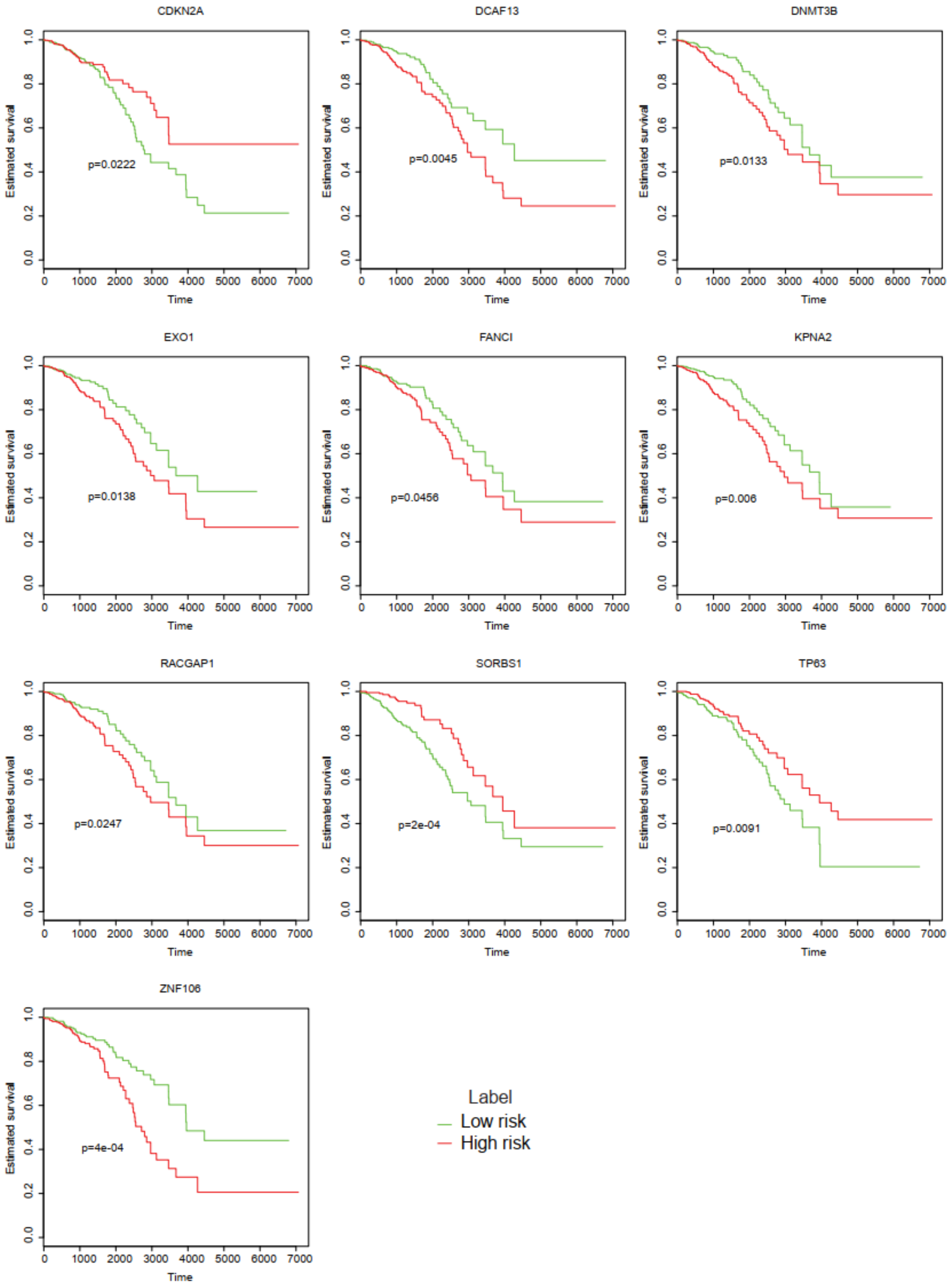

Figure S4. Kaplan-Meier plots of survival for the 10 RBPs.

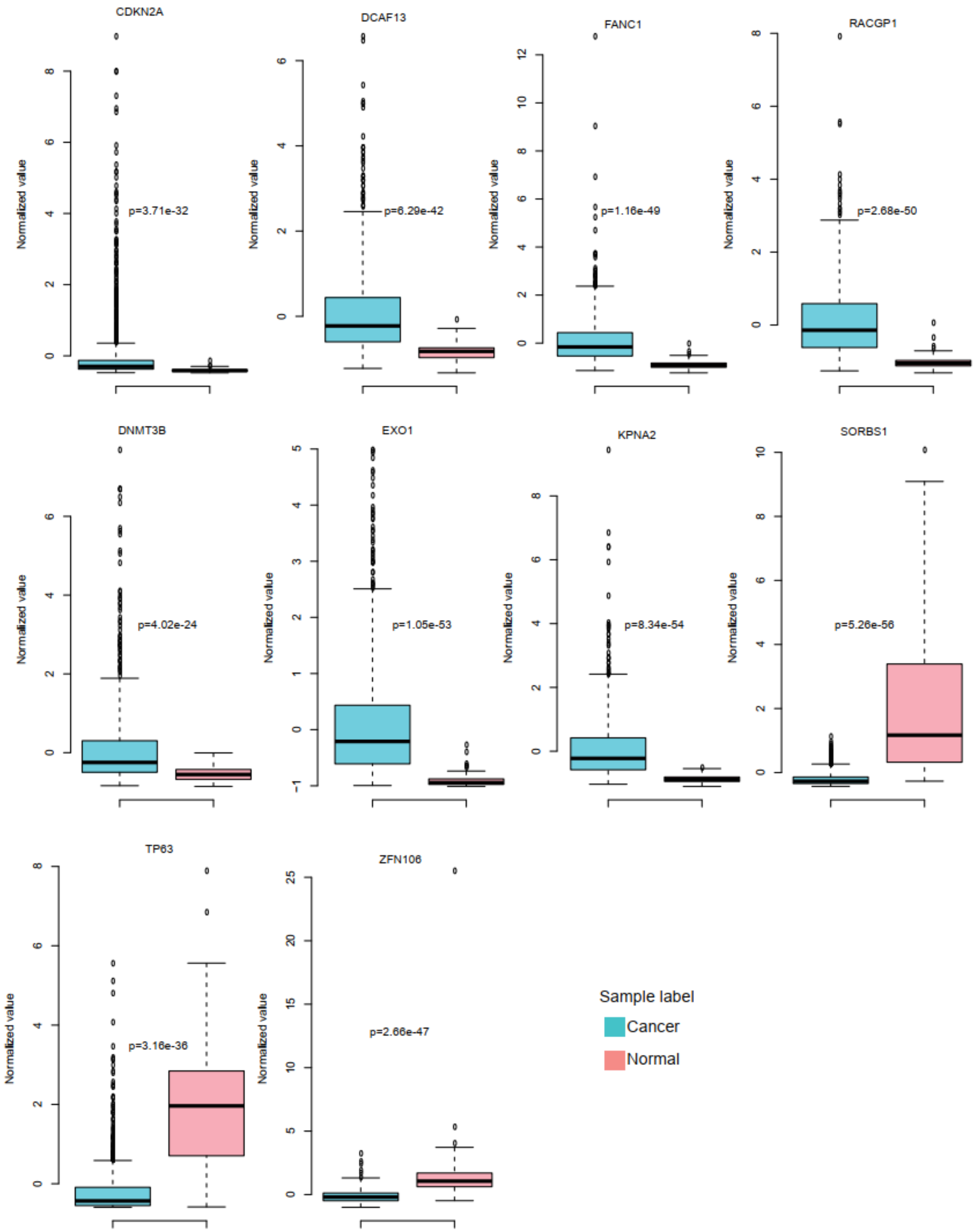

Figure S5. The expression patterns of the 10 RBPs in normal and cancer samples.
